# Multilevel Analysis of Women's Perceptions of Community‐Based Health Insurance on Healthcare Access in Sidama Region, Ethiopia: A Community‐Based Cross‐Sectional Study

**DOI:** 10.1002/hsr2.73097

**Published:** 2026-08-26

**Authors:** Kare Chawicha Debessa, Amanuel Yoseph

**Affiliations:** ^1^ School of Public Health, College of Medicine and Health Sciences Hawassa University Hawassa Ethiopia; ^2^ School of Public Health University of Saskatchewan Saskatoon Canada; ^3^ Department of Epidemiology and Biostatistics, Faculty of Health Sciences Western University London Canada

**Keywords:** community‐based health insurance, Ethiopia, healthcare access, multilevel analysis, women's perceptions

## Abstract

**Background and Aims:**

Access to healthcare remains a major challenge in low‐ and middle‐income countries, including Ethiopia, where financial and geographic barriers limit service utilization. Community‐Based Health Insurance (CBHI) has been introduced to enhance financial protection and improve healthcare access. This study examined factors associated with women's perceptions of CBHI in improving healthcare access in the Sidama Region, Southern Ethiopia, in 2024.

**Methods:**

A community‐based cross‐sectional study was conducted among women using a multistage stratified sampling approach. Data were collected through face‐to‐face interviews using the KoBoTool platform. Multilevel logistic regression analysis using Stata 18 examined individual‐ and community‐level determinants of women's perceptions of healthcare access. Adjusted odds ratios (AORs) with 95% confidence intervals were reported. Statistical significance was set at *p* < 0.05.

**Results:**

Of 845 women sampled, 835 (98.8%) were interviewed. Women residing in low‐poverty communities had significantly higher odds of perceiving improved access compared to those in high‐poverty areas (AOR = 4.6; 95% CI: 1.8–9.1). Rural residents were less likely to report improvements than urban residents (AOR = 0.37; 95% CI: 0.18–0.68). Recent health facility visits increased positive perceptions more than fourfold (AOR = 4.1; 95% CI: 1.9–8.2), whereas missed visits reduced the likelihood by 45% (AOR = 0.55; 95% CI: 0.34–0.88). Perceived inadequacy of the CBHI package and unfair contributions were associated with substantially lower odds of positive perception. Acceptance of CBHI emerged as the strongest predictor (AOR = 6.3; 95% CI: 2.1–13.6).

**Conclusion:**

Women's perceptions of CBHI are shaped by socioeconomic conditions, residence, healthcare utilization, and views on fairness and adequacy. Strengthening equitable and culturally responsive CBHI implementation may enhance healthcare access in Ethiopia and similar settings.

## 1 Introduction

Access to quality healthcare remains a major challenge worldwide. Many countries struggle to ensure fair access for all. In low‐ and middle‐income countries, high out‐of‐pocket payments create heavy financial burdens. These costs affect vulnerable groups the most, especially women, who often have limited control over household finances [1]. Paying directly for care can prevent people from seeking treatment. It also slows progress toward Universal Health Coverage (UHC). UHC ensures that everyone can use the health services they need without facing financial hardship. Achieving this goal requires strong, sustainable financing systems that protect households from catastrophic health costs [1].

Community‐based health insurance (CBHI) is one way countries like Ethiopia move toward UHC. CBHI is voluntary, locally managed, and designed to protect poor households. Unlike Social Health Insurance, which is mandatory for formal‐sector workers, or private insurance, which is profit‐oriented, CBHI is community‐driven [2]. In Ethiopia, it was introduced in 2011 as part of broader health financing reforms. Premiums are set affordably, often 200–400 Birr per year, about 5%–10% of the minimum monthly wage. Poorer households may pay less or be exempted [3]. The benefit package usually covers basic outpatient and inpatient services at public facilities. While not comprehensive, CBHI protects against common illnesses that could otherwise cause catastrophic costs [4].

Across sub‐Saharan Africa, CBHI has become an important strategy to reduce financial hardship and expand healthcare access [5]. Rwanda provides a successful example, with high coverage driven by political commitment. Other countries face persistent challenges, including financial sustainability, weak administrative capacity, and inconsistent service quality [6]. In Ethiopia, the program has expanded with government support, particularly targeting informal‐sector and marginalized households. Premium contributions are linked to income, which ensures fairness, though affordability and service quality remain concerns [7].

Women's participation in CBHI is influenced by education, autonomy, and household income [8]. Women are central to household health decisions but face unique barriers, including limited financial resources, cultural norms, and restricted mobility [9]. In the Sidama Region, Central Zone, CBHI enrollment among women has increased, but their perceptions of service quality, provider attitudes, and health infrastructure remain underexplored [10]. Understanding these perceptions is essential for designing policies that meet women's needs.

This study draws on health insurance theory and risk aversion theory from health economics. These theories suggest that households seek insurance to avoid unexpected financial hardship. High out‐of‐pocket payments motivate enrollment in schemes like CBHI. Women are sensitive to affordability. To lower direct payments and to offer subsidized premiums for poorer households, CBHI influences women's decisions to seek healthcare. This framework helps explain how CBHI shapes women's perceptions of access, satisfaction, and continuity of care [11].

We also applied the Donabedian framework to examine healthcare quality. It considers structure (facilities, staff, equipment), process (provider–patient interactions), and outcomes (health, satisfaction, equity) [12]. These dimensions shape how women perceive care quality. Weak infrastructure or poor provider attitudes can reduce trust, while supportive processes and well‐resourced facilities improve satisfaction and reinforce positive outcomes [13].

A conceptual model links CBHI enrollment, socio‐demographic factors (education, autonomy, income), and perceived quality. Enrollment provides access. Moderators influence participation and perception. Perceived quality affects satisfaction, continued participation, and trust. Together, these frameworks capture both service features and social influences on women's experiences under CBHI [14].

Evidence from Sidama shows that 35% of women were enrolled in CBHI in 2024, a rate higher than regional and some national averages. Enrolled women reported better access, lower out‐of‐pocket spending, and higher satisfaction than uninsured peers [8]. Challenges remain, including inconsistent service quality and coverage below national targets. A 2023 pilot program introduced sliding‐scale premiums to improve fairness and affordability [15].

Most studies focus narrowly on enrollment and service use, often using methods that ignore hierarchical data. Few examine specific domains of healthcare quality or socio‐demographic moderators [16]. Without such insights, policymakers cannot design CBHI reforms that fully meet women's needs. This gap threatens the program's sustainability and effectiveness [17].

This study addresses the gap by examining women's perceptions of healthcare quality under CBHI in Sidama. It focuses on three domains: accessibility, satisfaction, and treatment outcomes. By situating CBHI in Ethiopia's health financing history and applying relevant frameworks, the study provides evidence to improve CBHI design. Findings contribute to equity, support UHC goals, and inform policies that strengthen financial protection while enhancing healthcare quality for women.

## 2 Methods

### 2.1 Study Setting

This study analyzed primary data from research conducted in the Central Zone of the Sidama Region, Ethiopia, a regional state established in June 2020 [18]. The zone comprises six districts and one town administration, with a total population of 956,967 [19]. Dale Woreda and Yirgalem City Administration, located approximately 45 km south of Hawassa, the regional capital, were selected as study sites [20]. These sites were purposively chosen to represent both urban and rural settings in the Central Zone of Sidama. Both areas have well‐established CBHI programs with sufficient enrollment, allowing us to examine women's perceptions across diverse socio‐demographic and geographic contexts. Selecting these sites ensured the study was feasible, relevant, and capable of generating findings that can inform local and regional health policy [21].

### 2.2 Study Design and Period

A community‐based cross‐sectional study was conducted from December 15, 2023, to January 12, 2024, following the Strengthening the Reporting of Observational Studies in Epidemiology (STROBE) checklist (Supplementary File 1).Source and study population

The source population comprised all women aged ≥ 18 years residing in Dale Woreda and Yirgalem City Administration. Inclusion required continuous residence for at least 1 year to ensure adequate exposure to local healthcare systems. Women unable to communicate due to illness or cognitive impairment, temporarily absent during data collection, recent visitors, or those unable to provide informed consent were excluded, in line with STROBE methodological and ethical standards [22].

### 2.3 Sample Size and Sampling Method

The sample size was calculated using OpenEpi 3, based on a prior CBHI coverage of 49% in Tigray [8, 23]. Using a 5% margin of error and 95% confidence level. Applying a design effect of 2 [24] to account for clustering produced 768, based on an intraclass correlation coefficient (ICC) of 0.01 [25]. Fourteen *kebeles* (the lowest administrative unit in Ethiopia) were selected to enhance statistical power and representativeness, maintaining design effect at 2 [26]. Allowing for a 10% non‐response rate, the final sample size was 845.

The study employed a multistage sampling approach. Yirgalem City and Dale Woreda were purposively selected based on their relevance to the study objectives. Within each Woreda, *kebeles* (the smallest administrative units) were randomly selected as clusters. Fourteen *kebeles* were included: nine from Dale Woreda (eight rural, one urban) and five from Yirgalem City Administration (three rural, two urban). Random selection minimized bias and enhanced representativeness.

The total sample was proportionally allocated to each *kebele* according to population size, with eligible women per *kebele* ranging from 873 to 2192. As full cluster sampling was not feasible, simple random sampling was applied to lists of eligible women obtained from community health records to achieve the required sample size. At the household level, simple random sampling was again used to select households. Data collectors approached selected households to identify eligible women, prioritizing women due to their central role in household health decisions. Where more than one eligible woman was present, one was selected using a lottery method to ensure fairness. Up to three contact attempts were made for initially unavailable households, and visit details were documented to maintain data quality and accountability.

### 2.4 Study Variables

Variable selection was guided by Health Insurance Theory and Donabedian's Framework to capture both financial protection and healthcare quality dimensions.

#### 2.4.1 Outcome Variable

The primary outcome was women's perception of whether CBHI improves healthcare access. Participants were asked whether they believed CBHI enhances access to healthcare services in their community. Responses were coded as 1 (“Yes”) and 0 (“No”). This binary measure reflects the theoretical premise that financial risk protection reduces barriers and facilitates timely care‐seeking.

#### 2.4.2 Individual‐Level Variables

Individual factors included socioeconomic status, demographic characteristics, and CBHI‐related variables such as enrollment status, premium affordability, benefit awareness, and women's financial autonomy. These variables align with Health Insurance Theory, which emphasizes affordability, equity, and the role of financial protection in shaping healthcare utilization.

#### 2.4.3 Community‐Level Variables

Community‐level factors comprised residence (urban/rural), women's literacy, community poverty, and autonomy. In line with Donabedian's framework, these variables reflect structural and contextual determinants that may influence how CBHI operates within healthcare systems and moderates access outcomes. Detailed variable definitions and measurements are provided in Supplementary File S2.

### 2.5 Data Collection and Quality Assurance

A structured, pre‐tested questionnaire (Supplementary File S3), adapted from validated sources [27, 28], was used for data collection. The questionnaire was translated into Sidaamu Afoo and subsequently back‐translated into English to ensure accuracy, consistency, and originality. A pretest involving 5% of the sample was conducted in Hitata *kebele*, resulting in refinement of the tool and demonstrating high internal consistency (Cronbach's α = 0.90). Data were collected through face‐to‐face interviews using the KoBoTool mobile platform by 25 trained health science graduates, under the supervision of five master level public health professionals [29]. Daily data quality checks were performed to ensure completeness and validity. Before analysis, the dataset underwent comprehensive preprocessing, including verification of coding accuracy, data cleaning, and management of missing data through exclusion when imputation was inappropriate. Outliers were examined and addressed based on predefined criteria to minimize bias [30].

### 2.6 Data Analysis

Variables were coded and categorized before analysis (Supplementary File S4). Descriptive statistics were reported as frequencies and percentages for categorical variables and means with standard deviations for continuous variables. wealth index derived via principal component analysis [31]. Multilevel logistic regression estimated adjusted odds ratios (AORs) with 95% confidence intervals (CIs). The ICC values > 5% justified multilevel modeling [32]. Variables with *p* < 0.25 in bivariable analysis and literature‐supported factors were included in multivariable models [33], with multicollinearity assessed using the variance inflation factor < 5 [34].

We employed a multilevel modeling approach to account for the hierarchical structure of the data, with individuals (Level 1) nested within *kebeles* (Level 2). Four models were estimated sequentially: an empty model without predictors to assess baseline variance between *kebeles*; a model including only individual‐level variables; a model including only *kebele*‐level variables; and a final model incorporating both individual‐ and *kebele*‐level factors. Random intercepts were specified to allow the average outcome to vary across *kebeles*.

Model fit was evaluated using the deviance statistic (−2 log‐likelihood), with lower values indicating better fit. Comparative performance across models was assessed using the akaike information criterion (AIC) and Bayesian information criterion (BIC), where smaller values denote superior model fit [35]. The proportional change in variance (PCV) was calculated to quantify the reduction in unexplained between‐*kebele* variance across successive models, and the ICC was used to estimate the proportion of total variance attributable to *kebele*‐level clustering.

### 2.7 Statistical Software and Subgroup Analysis

All statistical analyses were conducted using Stata version 18.0 (Stata Corp, College Station, TX, USA) [36]. Descriptive statistics and multilevel logistic regression models were performed to examine associations between individual‐ and community‐level factors and women's perceptions of CBHI. All statistical tests were two‐sided, with a pre‐specified significance level of α = 0.05. Abbreviations, symbols, and statistical terms are clearly defined, and all analyses were performed following the Statistical Analyses and Methods in the Published Literature (SAMPL) guidelines for transparency and reproducibility [37]. We conducted a subgroup analysis stratified by district and by place of residence (urban vs. rural) to explore potential variations across settings.

## 3 Results

### 3.1 Random Model Information and Model Fitness

The null model (Model 0) had an ICC of 0.4415, showing that 44% of the variance in women's perception of healthcare access was between *kebeles*, supporting multilevel modeling. Adding individual‐level factors (Model 1) reduced the ICC to 0.3489, while community‐level factors alone (Model 2) gave 0.4407. The full model (Model 3) produced the lowest ICC (0.1874), reflecting the greatest reduction in between‐*kebele* variance (Table 1).

**Table 1:** Intraclass correlation coefficient estimates for four multilevel models examining women's perceptions of healthcare access in the Sidama Region, Southern Ethiopia, 2024 (*n* = 835).

Model No.	Model description	Intraclass correlation coefficient	Std. Err.	95% Confidence interval
0	Null model (no predictors)	0.4415	0.1084	0.3154–0.8184
1	Individual‐level factors	0.3489	0.1474	0.2022–0.7237
2	Community‐level factors	0.4407	0.1181	0.3167–0.7493
3	Both individual‐ and community‐level factors (full)	0.1874	0.1531	0.0312–0.6232

The null model fit poorly (AIC = 734.04; BIC = 743.50), Model 1 improved fit (AIC = 386.74; BIC = 514.39), Model 2 performed worse (AIC = 712.98; BIC = 746.07), and the full model (Model 3) had the best fit (AIC = 386.23; BIC = 499.69), supporting inclusion of both individual‐ and community‐level predictors (Table 2).

**Table 2:** Model fit indices for four multilevel models assessing women's perceptions of healthcare access in the Sidama Region, Southern Ethiopia, 2024 (*n* = 835).

Model No.	Model description	*N*	Log likelihood (ll(model))	Degrees of freedom	AIC	BIC
0	Null model (no predictors)	835	−365.0211	2	734.0422	743.4971
1	Individual‐level factors	835	−166.3722	27	386.7443	514.385
2	Community‐level factors	835	−349.4881	7	712.9763	746.0683
3	Both individual‐ and community‐level factors (full)	835	−169.1174	24	386.2348	499.6932

Abbreviations: AIC: akaike information criterion; BIC, Bayesian information criterion.

### 3.2 Study Participants Characteristics

In 2024, 835 women from the Central Zone of Sidama Region, Ethiopia, were surveyed. Most lived in low‐poverty communities (57.3%), had low literacy (53.7%), and low community autonomy (51.6%). The majority resided in rural areas (57.9%) and male‐headed households (88.8%), with 60% employed.

Regarding CBHI perceptions, 67.4% reported package inadequacy, 42.3% perceived contributions as unfair, while 59.3% and 57.3% viewed decision‐making as transparent and inclusive, respectively. Acceptance of CBHI was high (63.5%), with 57.5% willing to advocate for it. Positive perceptions were also noted for drug availability (79.5%), quality improvement (82.2%), affordability (81.7%), health service use (78.2%), and financial protection (79.0%). Most participants could read and write (57.3%) and were married (93.7%) (Table 3).

**Table 3:** Key background characteristics of women in the Central Zone of Sidama Region, Southern Ethiopia, 2024 (*n* = 835).

Variable	Subcategory	Frequency	Percent (%)
Community‐level women's poverty	Low	478	57.3
	High	357	42.7
Community‐level women's literacy	High	387	46.3
	Low	448	53.7
Community‐level women's autonomy	High	431	51.6
	Low	404	48.4
Residence	Rural	484	57.9
	Urban	351	42.1
House head	Female	94	11.2
	Male	741	88.8
Employment	Not employed	334	40.0
	Employed	501	60.0
CBHI packages adequacy	Not adequate	563	67.4
	Adequate	272	32.6
CBHI premium contribution fairness	It is not fair	353	42.3
	It is fair	482	57.7
CBHI decisions transparency	not transparent	340	40.7
	It is transparent	495	59.3
CBHI decisions inclusive	includes sometimes	357	42.7
	Includes all times	478	57.3
CBHI promotion adequacy	It is not adequate	419	50.2
	It is adequate	416	49.8
CBHI strategy acceptable	Yes	530	63.5
	No	305	36.5
Advocate CBHI to others	No	355	42.5
	Yes	480	57.5
CBHI ensures the constant availability of drugs	low to high	664	79.5
	None	171	20.5
CBHI improves healthcare quality	low to high	686	82.2
	None	149	17.8
CBHI provides affordable healthcare	low to high	682	81.7
	None	153	18.3
CBHI ensures health consumption	low to high	653	78.2
	None	182	21.8
CBHI ensures financial protection	low to high	660	79.0
	None	175	21.0
Recoded educational status	Read and write	479	57.3
	Do not read and write	356	42.7
Recoded marital status	Married	782	93.7
	Others	53	6.3

Abbreviation: CBHI, Community‐based health insurance.

Continuous variables showed a mean age of 38.7 years [standard deviation (SD) = 14.1] and an average family size of 4.82 (SD = 1.47). Participants reported an average of 0.85 health facility visits (SD = 1.26). Walking to the nearest facility took a mean of 44.9 min (SD = 15.6), with waiting times averaging 62 min (SD = 25.4). Overall healthcare satisfaction was low and variable, with a mean score of 11.7 (SD = 17.9) (Table 4).

**Table 4:** Descriptive statistics of key continuous variables among women in the Central Zone of Sidama Region, Southern Ethiopia, 2024 (*n* = 835).

Continuous variables	Mean	Standard deviation (SD)
Age	38.7	14.1
Family size	4.82	1.47
Frequency of health facility visits	0.85	1.26
Distance to health facility	44.9	15.6
Waiting times at health facilities	62.0	25.4
Healthcare satisfaction	11.7	17.9

### 3.3 Women's Perceptions of CBHI and Healthcare Access

**Figure 1:**
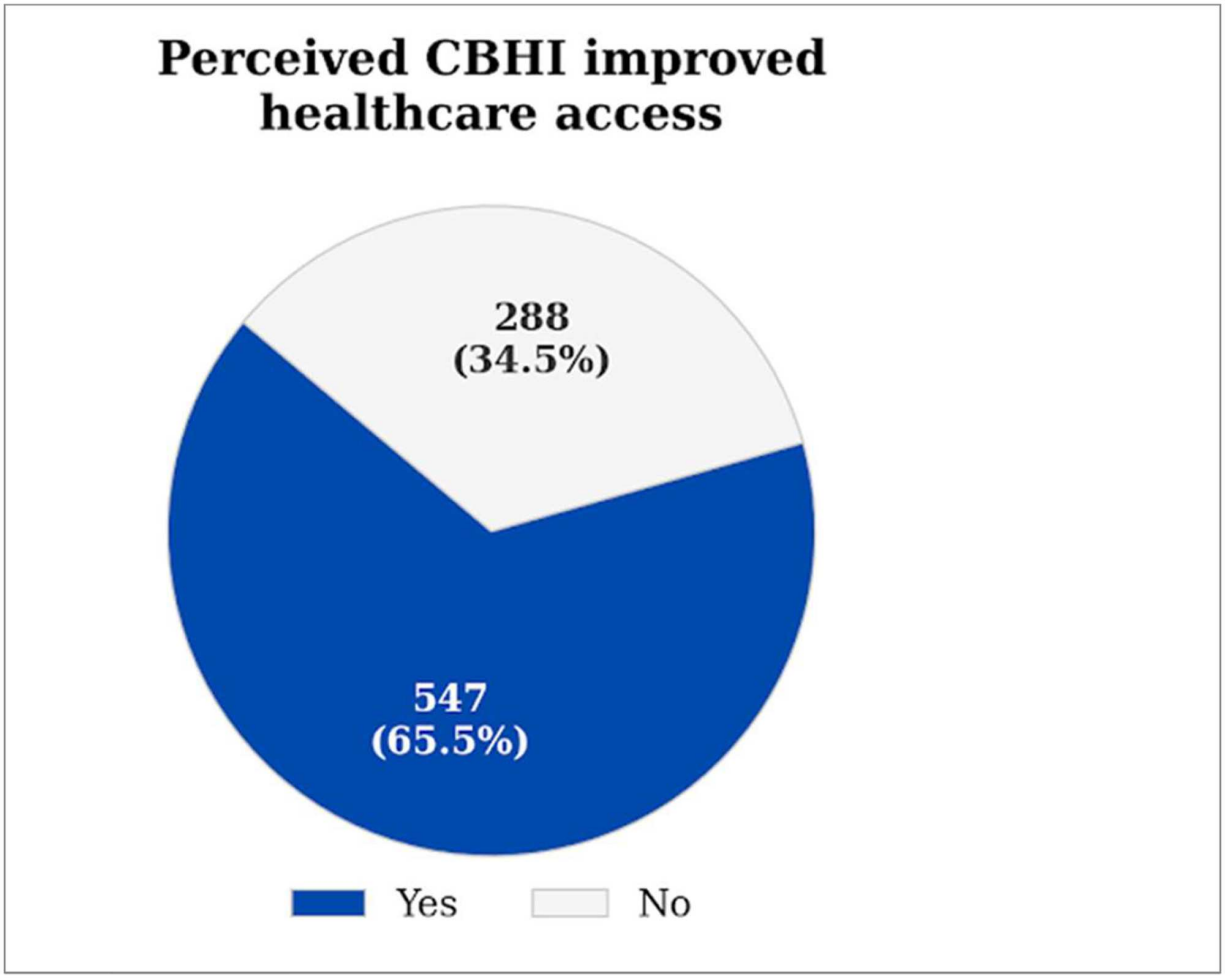
Women's perceptions of community‐based health insurance (CBHI) on healthcare access in the Central Zone of Sidama Region, Ethiopia, 2024 (*n* = 835).

Most women perceived that CBHI improved healthcare access. Of 835 participants, 547 (65.5%; 95% CI: 62.2–68.7) reported a positive perception, while 288 (34.5%; 95% CI: 31.3–37.8) did not reflect generally favorable views of the program (Figure 1).

Perceptions of CBHI's impact differed significantly between rural and urban women. Among rural participants, 269 (76.6%) reported improved access versus 82 (23.4%) who did not, while 278 (57.4%) urban women perceived improvement and 206 (42.6%) did not (χ^2^ = 33.19, *p* < 0.001). Rural women were more likely to perceive CBHI as beneficial, likely due to reduced financial barriers and limited local services, whereas urban women reported less impact where health facilities were more accessible (Table 5).

**Table 5:** Perceptions of community‐based health insurance (CBHI) on healthcare access by residence among women in the Central Zone, Sidama Region, Southern Ethiopia, 2024 (*n* = 835).

CBHI's perception of improved healthcare access	Area of residence		
	Rural (1)	Urban (2)	Total
0 = No improvement	82 (28.47%)	206 (71.53%)	288 (34.49%)
1 = Improved access	269 (49.18%)	278 (50.82%)	547 (65.51%)
Total	351 (42.04%)	484 (57.96%)	835 (100%)

*Note:* Pearson chi^2^ (1) = 33.19, *p* < 0.001 and CBHI: Community‐based health insurance.

### 3.4 Determinants of Women's CBHI Perceptions

Multilevel logistic regression identified individual‐ and community‐level determinants of women's perception that CBHI improved healthcare access. Women in low‐poverty communities were over four times more likely to perceive benefits than those in high‐poverty areas (AOR = 4.63; 95% CI: 1.8–9.1). Rural women had lower odds than urban women (AOR = 0.37; 95% CI: 0.18–0.68). Health facility visits increased positive perception (AOR = 4.10; 95% CI: 1.9–8.2), while missed visits reduced it (AOR = 0.55; 95% CI: 0.34–0.88). Perceptions of package adequacy (AOR = 0.29; 95% CI: 0.11–0.61) and contribution fairness (AOR = 0.25; 95% CI: 0.12–0.59) significantly affected perceptions. Acceptance of CBHI was the strongest determinant, with over sixfold higher odds of positive perception (AOR = 6.3; 95% CI: 2.1–13.6) (Table 6).

**Table 6:** Multilevel determinants of women's perceptions of community‐based health insurance on healthcare access in the Central Zone, Sidama Region, Southern Ethiopia, 2024 (*n* = 835).

Variables	Category	Perceived that the CBHI program improved access to healthcare		COR (95% CI)	AOR (95% CI)	*p* values
		No (%)	Yes (%)			
Community‐level women's poverty	Low	108 (29.4)	259 (70.6)	5.50 (1.12– 6.01)	4.63 (1.81–9.11)	*p* = 0.034*
	High	180 (38.5)	288 (61.5)	1	1	
Community‐level women's literacy	High	72 (18.3)	322 (81.7)	4.29 (3.13–5.89)	1.45 (0.51–4.14)	*p* = 0.778
	Low	216 (49.0)	225 (51.0)	1	1	
Community‐level women's autonomy	High	108 (25.1)	323 (74.9)	2.40 (1.79–3.22)	0.70 (0.33–1.45)	*p* = 0.861
	Low	180 (44.6)	224 (55.4)	1	1	
Residence	Rural	206 (42.6)	278 (57.4)	0.41 (0.30–0.56)	0.37 (0.18–0.68)	*p* = 0.025*
	Urban	82 (23.4)	269 (76.6)	1	1	
Ever visited a health facility	Yes	73 (21.2)	271 (78.8)	5.89 (2.11–7.96)	4.10 (1.91–8.20)	*p* = 0.007*
	No	215 (43.8)	276 (56.2)	1	1	
CBHI packages adequacy	Not adequate	276 (47.8)	301 (52.2)	0.55 (0.03–0.10)	0.29 (0.11–0.61)	*p* < 0.001**
	Adequate	12 (4.7)	246 (95.3)	1	1	
Premium contribution fairness	Not fair	272 (60.4)	178 (39.6)	0.43 (0.02–0.65)	0.25 (0.12–0.59)	*p* < 0.001**
	Fair	16 (4.2)	369 (95.8)	1	1	
CBHI decisions transparency	Not transparent	281 (49.7)	284 (50.3)	1.03 (0.01–1.06)	0.93 (0.26–3.32)	*p* = 0.835
	Transparent	7 (2.6)	263 (97.4)	1	1	
CBHI decisions are inclusive	Includes sometimes	283 (45.1)	345 (54.9)	0.63 (0.01–0.87)	0.31 (0.08–1.17)	*p* = 0.837
	Includes all times	5 (2.4)	202 (97.6)	1	1	
CBHI acceptable strategy	Yes	42 (7.9)	489 (92.1)	8.38 (5.26–21.58)	6.31 (2.10–13.6)	*p* < 0.001**
	No	246 (80.9)	58 (19.1)	1	1	
Advocate CBHI to others	No	277 (45.1)	337 (54.9)	0.06 (0.03–0.12)	0.78 (0.29–2.11)	*p* = 0.605
	Yes	11 (5.0)	210 (95.0)	1	1	
CBHI ensures constant drug availability	Low to high	157 (23.7)	506 (76.3)	10.30 (6.95–15.27)	2.78 (0.94–8.18)	*p* = 0.432
	None	131 (76.2)	41 (23.8)	1	1	
CBHI improves the quality of services	Low to high	164 (23.9)	522 (76.1)	15.79 (9.93–25.11)	2.39 (0.59–9.68)	*p* = 0.178
	None	124 (83.2)	25 (16.8)	1	1	
CBHI provides affordable healthcare	Low to high	166 (24.4)	515 (75.6)	11.83 (7.72–18.12)	0.75 (0.19–2.92)	*p* = 0.754
	None	122 (79.2)	32 (20.8)	1	1	
CBHI ensures health consumption	Low to high	158 (24.2)	494 (75.8)	7.67 (5.32–11.06)	1.58 (0.43–5.72)	*p* = 0.532
	None	130 (71.0)	53 (29.0)	1	1	
CBHI ensures financial protection	Low to high	158 (23.9)	502 (76.1)	9.18 (6.26–13.47)	2.13 (0.68–6.66)	*p* = 0.392
	None	130 (74.3)	45 (25.7)	1	1	
Recoded education	Literate	128 (27.4)	340 (72.6)	2.05 (1.54–2.74)	1.08 (0.57–2.04)	*p* = 0.621
	Illiterate	160 (43.6)	207 (56.4)	1	1	
Women autonomy	Autonomous	104 (27.9)	269 (72.1)	0.58 (0.44–0.78)	0.52 (0.21–1.32)	*p* = 0.290
	No autonomous	184 (39.8)	278 (60.2)	1	1	
Distance to health facility ©				1.00 (1.00–1.01)	1.00 (0.99–1.01)	*p* = 0.194
Waiting times at health facility ©				1.00 (1.00–1.01)	1.00 (1.00–1.00)	*p* = 0.643
Frequency of health facility visits ©				1.28 (1.13–1.46)	0.55 (0.34–0.88)	*p* = 0.042*
Family size ©				0.99 (0.90–1.09)	0.91 (0.73–1.12)	*p* = 0.732

Abbreviations: AOR, adjusted odds ratio; CI, confidence interval; ©, continuous variable; COR, crude odds ratio; 1, reference group; CBHI, Community‐based health insurance.

* Significant association (*p* < 0.05).

** Highly significant association (*p* < 0.01).

## 4 Discussion

Using a multilevel approach, our study showed that both individual‐ and community‐level factors shape women's perceptions of CBHI. Household poverty, healthcare visits, and program awareness had the strongest effects, while neighborhood poverty, literacy, and autonomy also contributed. Despite CBHI's pro‐poor design, women in high‐poverty areas reported lower perceived benefits, likely due to enrollment barriers and limited awareness. Findings highlight the interplay between personal and contextual factors, emphasizing the need for targeted strategies to enhance CBHI's reach and impact.

Women from low‐poverty communities were significantly more likely to perceive CBHI as improving healthcare access. This is consistent with evidence from sub‐Saharan Africa, where higher socioeconomic status (SES) communities' benefit from better infrastructure, health literacy, and social capital, facilitating awareness and use of services [38, 39]. Reduced strain on facilities in these areas also supports positive perceptions. These findings underscore how community SES shapes both actual and perceived CBHI benefits, highlighting persistent barriers to equitable healthcare [40].

Rural residence substantially reduced the likelihood of perceiving improved healthcare access through CBHI, reflecting persistent rural‐urban disparities. Rural women face shortages of trained staff, medicines, and adequately equipped facilities, compounded by poor transport, limited health information, and lower literacy [41, 42]. These challenges constrain CBHI's reach, highlighting the urgent need for targeted system strengthening and infrastructure investments to achieve equitable access [43]. Although CBHI seeks to improve access, systemic deficits in rural areas limit its perceived benefits [3]. Reduced exposure to formal healthcare also lowers awareness and positive perceptions among rural women, consistent with qualitative evidence from Ethiopia and similar contexts [16, 44].

This study further identified health facility visitation as a strong positive predictor of perceived improvement in healthcare access. Women who had recent encounters with health services under the CBHI scheme were more likely to report tangible benefits, reflecting a direct experiential validation of the program's effectiveness [8]. This finding aligns with behavioral health theories emphasizing that personal experience with healthcare services shapes trust, satisfaction, and subsequent engagement [45].

In contrast, frequent non‐healthcare visitation was inversely associated with perceptions of CBHI benefits, indicating a bidirectional and potentially reinforcing feedback loop whereby utilization enhances trust and perceived value, which in turn motivates further service use [46]. These dynamics highlight the importance of demand‐side factors, including health‐seeking behavior and perceived service quality, in shaping program success [47]. Strategies to encourage healthcare utilization, particularly among marginalized or disengaged groups, are essential for optimizing CBHI's benefits.

Perceptions of CBHI's adequacy and fairness strongly influenced women's views of its effectiveness. Women who considered benefit packages insufficient or contribution mechanisms unfair were far less likely to perceive improved healthcare access. This aligns with prior evidence emphasizing that well‐designed benefits, equitable cost‐sharing, and transparent, inclusive governance are critical for participant satisfaction and program legitimacy [48]. Acceptance of the CBHI strategy itself, reflecting sociocultural fit and trust, was the strongest positive determinant, highlighting that community buy‐in and culturally sensitive implementation are essential for health insurance schemes to achieve meaningful impact [49].

When beneficiaries perceive CBHI as legitimate, responsive, and culturally appropriate, they are more likely to use services and report satisfaction, reinforcing positive perceptions. This aligns with implementation science, which highlights acceptance and contextual fit as essential for uptake and sustainability. Adapting CBHI to local cultural norms, engaging community leaders, and incorporating beneficiary feedback are therefore crucial strategies to strengthen program effectiveness and community trust [50].

### 4.1 Strengths and Limitations of the Study

This study has several strengths. A large, representative sample of women from the Central Zone of Sidama Region supports generalizability. The inclusion of both individual‐ and community‐level factors enabled a multilevel analysis, capturing social, economic, and program‐related determinants of perceptions. Adjusted odds ratios controlled for confounders, providing reliable estimates. Detailed assessment of CBHI‐specific perceptions (e.g., adequacy, fairness, transparency) adds granularity often absent in broader evaluations. Focusing on women, central to household health decisions, enhances public health relevance. Findings align with previous studies, strengthening external validity.

However, there are limitations. The cross‐sectional design precludes causal inference; positive perceptions may influence healthcare utilization as much as the reverse. Self‐reported data may be affected by recall or social desirability bias. Quantitative methods limit insight into deeper social and cultural factors, highlighting the value of future qualitative studies. Certain influential variables, such as detailed health status and provider perspectives, were not captured, so caution is needed when generalizing beyond this region. Lastly, we acknowledge that, in the absence of actual enrollment data, women's perceptions of CBHI may not fully reflect real uptake. Future studies should investigate how these perceptions translate into actual enrollment to better understand the scheme's impact and effectiveness.

### 4.2 Implications for Policy and Practice

The findings highlight the need for targeted strategies to improve CBHI effectiveness in Ethiopia and similar low‐income countries. Socioeconomic disparities should be addressed through subsidies or differentiated premiums to enhance access and perceived benefits among poorer communities. Rural health systems require strengthening via staffing, infrastructure, medicine supply, and transportation to ensure coverage translates into actual service use.

CBHI program improvements are essential. Transparent, inclusive decision‐making, fair contributions, and periodic review of benefit packages can build trust and ensure perceived adequacy. Engaging communities in program design fosters ownership and cultural alignment, supporting higher enrollment and satisfaction.

Health promotion and education should emphasize CBHI benefits, particularly for rural and infrequent healthcare users, while local health workers should be trained to communicate advantages and address concerns, boosting program visibility and uptake.

## 5 Conclusion

This study highlights how social, economic, and programmatic factors shape women's perceptions of CBHI in the Sidama Region. Key determinants include socioeconomic status, rural residence, healthcare use, and views on fairness and cultural fit. Strengthening rural health infrastructure, ensuring transparent and equitable program governance, and culturally tailoring CBHI strategies are essential to improve both perceived and actual benefits. Future longitudinal and mixed‐methods research is needed to further explore these dynamics and guide sustainable policies toward universal health coverage in Ethiopia.

## Author Contributions

**Kare Chawicha Debessa:** conceptualization, investigation, funding acquisition, writing – original draft, methodology, validation, visualization, writing – review and editing, software, formal analysis, project administration, data curation, supervision, resources. **Amanuel Yoseph:** conceptualization, investigation, funding acquisition, writing – original draft, writing – review and editing, visualization, validation, methodology, software, formal analysis, project administration, data curation, supervision, resources.

## Ethics Statement

The study adhered to the Declaration of Helsinki. Ethical approval was obtained from the Institutional Review Board of the College of Medicine and Health Sciences, Hawassa University (IRB/021/16, 22/11/2023). Support letters were secured from the School of Public Health and the Sidama Region Health Bureau and shared with local health authorities. All participants received full information on the study's purpose, procedures, risks, benefits, and confidentiality. Written informed consent was obtained, and interviews were conducted in private locations to ensure comfort and privacy.

## Conflicts of Interest

The authors declare no conflicts of interest.

## Supporting information


Supporting File 1

Supporting File 2

Supporting File 3

Supporting File 4

## Data Availability

The data that supports the findings of this study are available in the supporting material of this article.
