# Supplementary material for: Multilevel Analysis of Women's Perceptions of Community‐Based Health Insurance on Healthcare Access in Sidama Region, Ethiopia: A Community‐Based Cross‐Sectional Study
Source: Health Sci Rep. 2026 Aug 26;9(9):e73097. doi: 10.1002/hsr2.73097 (PMC13508016; doi:10.1002/hsr2.73097)
Supplement: Supplementary file 1 [file HSR2-9-e73097-s002.docx]

**Independent variables measurement**

The term **age** denotes the chronological age of women as reported at the time of data collection.

**Family size** is defined as the total number of individuals residing in a household at the time of data collection.

**CBHI enrollment status** refers specifically to whether an individual is currently enrolled as a member of the CBHI scheme or not. It is recorded as a dichotomous variable: either "enrolled (yes)" or "not enrolled (no).". This clearly distinguishes CBHI members from non-members. It is coded as **1 = yes (member)** and **0 = no (non-member, reference category)**, distinguishing insured from uninsured households for analytical evaluation.

**Women's autonomy** refers to the proportion of women who were able to make decisions regarding participation in community-based health insurance (CBHI) and other livelihood matters, either independently or jointly with their husbands. Conversely, women who lacked this ability, whether independently or collaboratively, were classified as Un-autonomous when decision-making authority rested solely with male household members.

**Community-level women's autonomy** serves as a proxy measure for assessing women's decision-making power within the *kebel*e. Communities were categorized as autonomous (high community-level autonomy) or nonautonomous (low community-level autonomy) based on women's involvement in decision-making related to livelihood issues at the community or *kebele* level.

A community was considered to exhibit women's autonomy if over 50% of women participated in decision-making processes - either independently or in conjunction with their husbands - at the *kebele* level. If this threshold is not met, indicating that fewer than 50% of women are engaged in decision-making, the community is classified as non-autonomous or low community-level autonomy. Coding for this variable was **1 = high autonomy**, indicating greater female engagement and influence, and **0 = low autonomy (reference category)**, indicating limited participation.

**Community-level poverty** was assessed by calculating the aggregated percentage of households within a *kebele* that belong to the poorest and poorer quintiles based on the woman participant data. If this percentage exceeds 50%, the community is classified as having a "high community-level of poverty”; otherwise, it is designated as "low community-level poverty." This variable was coded as **0 = low poverty (less poverty)** and **1 = high poverty (reference category, more poverty)**, enabling comparison between communities with lower versus higher prevalence of women’s poverty.

**Community-level women's literacy** was determined by the proportion of women within a *kebele* who have achieved at least a primary level of education. If more than 50% of women have completed at least primary education, the community is classified as having a "high community-level literacy or literate" community; otherwise, it is categorized as "low community-level literacy or illiterate." Literate communities were coded as **1 = high literacy (reference category)**, whereas communities with lower literacy rates are coded as **0 = low literacy**, allowing analysis of how literacy levels influence outcomes.

**Wealth index:** This variable represents an aggregate measure of household assets, determined through principal component analysis (PCA). It categorizes households into five socioeconomic groups, from poorest to richest. Asset components assessed included livestock, crop production, infrastructure (radio, modern bed, mattress, phone, water pump, and modern stove), latrine facilities, housing conditions (number of rooms, roof, and floor), and total farm size.

PCA was employed to reduce dimensionality in the large data set. Variables possessed by less than 5% or more than 95% of households were excluded to ensure meaningful variation. Additionally, variables failing to meet assumptions, such as a Kaiser-Meyer-Olkin (KMO) measure below 0.5, communalities under 0.5, or complex structures with high cross-loadings (>0.4 on multiple components), were removed. The final wealth index scores were divided into five ordinal categories: 0 = poorest, 1 = poor, 2 = middle, 3 = rich, and 4 = richest. This classification effectively differentiates households by socioeconomic status.

**Healthcare Satisfaction:** Health service satisfaction is a composite measure that assesses patients' satisfaction in the past twelve months with healthcare services through their responses to 15 specific questions. Each question is rated on a five-point Likert scale, with 1 representing "strongly dissatisfied" and 5 representing "strongly satisfied."

The overall satisfaction score is derived by summing the individual ratings, resulting in a total score that ranges from 15 (indicating all responses are "strongly dissatisfied") to 75 (indicating all are "strongly satisfied"). This quantitative method enables the assessment of health service satisfaction, specifically among women’s groups.

**CBHI benefit packages adequacy** measures the perceived sufficiency of the health services and benefits covered by CBHI as reported by the women via standardized surveys. This variable was coded **1 = adequate (reference category)** when members perceive the benefits meet their needs effectively, and **0 = not adequate** if they feel the benefits were insufficient.

**CBHI decisions transparency** assesses how open and clear the decision-making processes of the CBHI scheme were perceived by members. It is coded **1 = transparent (reference category)** when decisions are deemed open and communicated, and **0 = not transparent** when decisions are perceived as opaque or unclear.

**CBHI decisions inclusively** capture the level of participation by diverse community members, including women and marginalized groups, in CBHI governance and decisions. This variable was coded **1 = always included (reference category)** when participation is comprehensive, and **0 = includes sometimes** when involvement was partial or irregular.

**CBHI advocacy adequacy** reflects community perceptions on the sufficiency and effectiveness of promotional efforts to encourage CBHI enrollment and awareness. It was coded **1 = adequate (reference category)** when promotion is considered sufficient and effective, and **0 = not adequate** when it was considered insufficient.

**CBHI strategy acceptance** addresses the community or member-level acceptance of the CBHI program’s design and implementation. This variable was coded **1 = yes (accepted)** and **0 = no (not accepted, reference category)** to capture positive versus negative attitudes toward the scheme strategy.

**advocate CBHI to others,** and record whether members actively promote CBHI enrollment to others. It was coded as **1 = yes (reference category)** when members endorse CBHI, and **0 = no** if they do not engage in advocacy or promotional activities.

**CBHI ensures constant drug availability** measures beneficiaries’ perceptions regarding the reliability of essential drug supplies at CBHI-affiliated facilities. Coding was **0 = no availability (reference category)** when drugs are often unavailable, and **1 = low to high availability** when drug availability was perceived as consistently adequate or improving.

**CBHI provides affordable healthcare** evaluates whether healthcare services under CBHI are perceived as financially affordable by members. It was coded **0 = no affordability (reference category)** when healthcare costs remain burdensome, and **1 = low to high affordability** when costs were manageable or effectively subsidized.

**CBHI ensures health consumption** reflects the effectiveness of CBHI in increasing members’ utilization of healthcare services. It was coded **0 = no consumption (reference category)** when utilization is inadequate or unchanged, and **1 = low to high consumption** when CBHI contributes to greater service use.

**CBHI ensures financial protection** and assesses the degree to which CBHI shields households from catastrophic health expenditures or financial hardship. This variable was coded **0 = no financial protection (reference category)** when households remain vulnerable, and **1 = low to high financial protection** when CBHI effectively reduces financial risk.
