# Supplementary material for: Multilevel Analysis of Women's Perceptions of Community‐Based Health Insurance on Healthcare Access in Sidama Region, Ethiopia: A Community‐Based Cross‐Sectional Study
Source: Health Sci Rep. 2026 Aug 26;9(9):e73097. doi: 10.1002/hsr2.73097 (PMC13508016; doi:10.1002/hsr2.73097)
Supplement: Supplementary file 2 [file HSR2-9-e73097-s003.docx]

**Participant information sheet**

Good morning/afternoon.

My name is---------------. I am from Hawassa University and researching community-based health insurance among women in Sidama Regional State.

Objective: to determine community-based health insurance prevalence and its determinants among women in Sidama National Regional State, Ethiopia.

You are selected randomly as a potential participant during this study as a subject.

**Potential risks:** There will be no potential risk that may cause any harm to study participants.

**Benefits:** There are no financial benefits associated with this study. However, by taking part in this study, you may contribute to improving the performance of community-based health insurance among women groups.

**Confidentiality:** All information given by you will be kept strictly confidential. Your participation shall be voluntary and not obligated to answer any question that you do not wish to answer. If you feel any discomfort with the questionnaire, please feel free to drop it at any time. This questionnaire will take approximately 45 minutes.

Are you willing to participate in the study?

1- Yes	2 - No

If the answer is yes, thanks! Continue with the interview.

If the answer is no, Thanks! Never force or reinforce study participants in the study

**Written consent from participants**

Hawassa University

I have understood all about the objective and the process of the study. My participation is voluntary and not obligated to answer any question that I do not know or do not wish to answer. I also understood that all information given to me will be kept strictly confidential. Therefore, I am willing to participate in this study.

Study participant sign _____________________________ date _____________

Data collector name: _________________________________________Signature _________

Date of interview ----------- Month --------------- /2016 E. C.

Supervisor’s Name ___________________________________________ Signature________

Checked date________________ /2016 E.C.

Contact Address of the Principal investigator

Name: Kare Chawicha

E-Mail: [kare.debessa@gmail.com](mailto:abigiatenaw@gmail.com)

Cell-Phone: +251900505922

Annex 4: English Version questionnaires

Hawassa University, School of Public Health

This questionnaire is designed to census community-based health insurance among women from selected woredas in Sidama Regional State, Ethiopia.

Woreda: ______________ Kebele code___________ Household code ______________

Section I: Background characteristics of the study participants

| S. No | Question | Response |
| --- | --- | --- |
| 1.1 | How old are you? | ____years (in completed year) |
| 1.2 | What is your religion? | 1. Orthodox  2. Catholic  3. Protestant  4. Muslim  5. Traditional  6. Other (Specify)___________ |
| 1.3 | What is your ethnic group | 1. Sidama  2. Amhara  3. Wolaita  4. Gurage  5. Siltie  6. Others (specify)____________ |
| 1.4 | What is your marital status | 1. Never married  2. Married  3. Living together  4. Divorced/separated  5. Widowed |
| 1.5 | Residence | 1. Urban  2. Rural |
| 1.6 | Kebele | Mention: __________________ |
| 1.7 | With whom you are living now?  (Circle all apply) | 1. Husband  2. Family member  3. Alone  4. Relatives  5. Friends or peers  6. Others (specify)___________ |
| 1.8 | Who makes final decisions at a household level? | 1. husband  2. wife  3. Both |
| 1.9 | Who is the household head | 1. male  2. female |
| 1.10 | What is the number of your family size? | 1. One  2. Two  3. Three  4. Four  5. Five  6. Six  7. Seven and above |
| 1.11 | What is your educational level? | 1. Unable to read and write  2. Primary education  3. Secondary education  4. Diploma and above |
| 1.12 | What is your employment status? | 1.Employed  2.Not employed |
| 1.13 | What is your occupation? | 1. Government employee  2. Private  3. Domestic worker  4. Self employed  5. Unskilled manual  6. Unpaid family member  7. non-governmental organization  8. Other (specify)____________ |

Section II: Community-based health insurance-related questions

| S. No | Question | Response |
| --- | --- | --- |
| 2.1 | Are you currently a member of the CBHI? | 1. Yes, if yes, skip to question 2.5  2. No |
| 2.2 | If you are not a member, what is (are the reasons (s)? Select all that apply | 1. Lack of knowledge 2. Lack of money 3. Because of a bad image 4. Some inconveniences 5. Lack of trust 6. Because I can afford health costs without difficulties |
| 2.3 | Would you like to join the CBHI scheme in the future? | 1. Yes 2. No 3. Not decided yet |
| 2.4 | If yes, how certain to join the scheme? | 1. Certainly join 2. I do not know when to join |
| 2.5 | If you are a member, for how long have you been a member of the CBHI? | 1. up to one year  2. up to two years  3. up to three years  4. up to four years  5. up to five years  6. up to six or more years |
| 2.6 | Are you contributing a premium to the scheme regularly? | 1. Regularly  2. With some interruptions  3. Ceased contribution |
| 2.7 | Have you ever dropped out of CBHI? | 1. Yes  2.No |
| 2.8 | If yes, what were the reasons for the dropouts? More than one answer may apply | 1. Affordability (lack of money)  2. Lack of medications and drugs at the health facility  3. Lack of laboratory services  4. Bad perception of the scheme  5. Poor behavior of the staff  6. Poor service quality  7. Lack of transparency in the scheme  8. Lack of inclusiveness in decision-making  9. Because I got an alternate health financing opportunity |
| 2.9 | When did you drop out? Please specify the year in the Ethiopian calendar | 1.2014  2.2013  3.2012  4.2011  5.2010 and before |
| 2.10 | Have you ever been selected as a committee member of the CBHI? | 1. Yes  2.No |
| 2.11 | Who is the final decision-maker of the scheme at the woreda level? | 1. Woreda administration only  2. The Woreda scheme committee  3. The general assembly of the scheme  4. The regional health bureau |
| 2.12 | Have you or any of the family members ever been sick during the last 12 months? | 1. Yes  2. No |
| 2.13 | Have you or any one of your family members visited a health facility during the last 12 months? | 1. Yes  2.No |
| 2.14 | If yes, how frequently have you and/ or your family visited? | ----------------times |
| 2.15 | Which institution have you visited? | 1. Government health facility  2. Private health facility  3. Non- governmental health facility  4. Traditional healers |
| 2.16 | If you or your family members were treated in any one of the above facilities, what was the treatment outcome? | 1. Cured  2. Still sick  3. disability  4. On follow-up  5. death |
| 2.17 | Approximately how far is the nearest health facility on walking from your home? | 1. Half an hour  2. half to an hour  3. more than one hour |
| 2.18 | On average, how long do you or your family members wait at a health facility (in hours)? | 1. Less than 30 minutes  2. 30 minutes to an hour  3. One to two hours  4. Two to three hours  5. Three to three hours  6. More than four hours |
| 2.19 | Accessible and convenient to reach out to a health facility | 1. Strongly dissatisfied 2. Dissatisfied 3. Neutral 4. Satisfied 5. Strongly satisfied |
| 2.20 | The reception at the card room | 1. Strongly dissatisfied 2. Dissatisfied 3. Neutral 4. Satisfied 5. Strongly satisfied |
| 2.21 | The care provided as outpatient services | 1. Strongly dissatisfied 2. Dissatisfied 3. Neutral 4. Satisfied 5. Strongly satisfied |
| 2.22 | The care provided as inpatient services | 1. Strongly dissatisfied 2. Dissatisfied 3. Neutral 4. Satisfied 5. Strongly satisfied |
| 2.23 | With the laboratory services | 1. Strongly dissatisfied 2. Dissatisfied 3. Neutral 4. Satisfied 5. Strongly satisfied |
| 2.24 | Explanation of diagnosis and treatment options | 1. Strongly dissatisfied 2. Dissatisfied 3. Neutral 4. Satisfied 5. Strongly satisfied |
| 2.25 | Treatment with respect and dignity | 1. Strongly dissatisfied 2. Dissatisfied 3. Neutral 4. Satisfied 5. Strongly satisfied |
| 2.26 | Health facility cleanliness and neatness | 1. Strongly dissatisfied 2. Dissatisfied 3. Neutral 4. Satisfied 5. Strongly satisfied |
| 2.27 | Privacy and confidentiality maintained | 1. Strongly dissatisfied 2. Dissatisfied 3. Neutral 4. Satisfied 5. Strongly satisfied |
| 2.28 | Information provided on medication and its side effects | 1. Strongly dissatisfied 2. Dissatisfied 3. Neutral 4. Satisfied 5. Strongly satisfied |
| 2.29 | With scheduling and waiting times | 1. Strongly dissatisfied 2. Dissatisfied 3. Neutral 4. Satisfied 5. Strongly satisfied |
| 2.30 | Participation in treatment decisions | 1. Strongly dissatisfied 2. Dissatisfied 3. Neutral 4. Satisfied 5. Strongly satisfied |
| 2.31 | Management of pain effectively | 1. Strongly dissatisfied 2. Dissatisfied 3. Neutral 4. Satisfied 5. Strongly satisfied |
| 2.32 | The overall attitude of healthcare providers | 1. Strongly dissatisfied 2. Dissatisfied 3. Neutral 4. Satisfied 5. Strongly satisfied |
| 2.33 | The overall quality of healthcare received | 1. Strongly dissatisfied 2. Dissatisfied 3. Neutral 4. Satisfied 5. Strongly satisfied |
| 2.34 | Recommend this health facility to others | 1. Completely disagree 2. Disagree 3. Neutral 4. Agree   Completely agree |
| 2.35 | What experience do you have with ambulance services? | 1. Readily available at any time  2. Respond sometime  3. Ambulance service is not available at all |
| 2.36 | Is an adequate water source available in the health facility? | 1. Yes  2. No  3. I don’t know |
| 2.37 | Is on-grid power connected to the health facility? | 1. Yes  2.No  3. I don’t know |
| 2.38 | Which critical services did you lack from the health facility? (More than one answer may apply) | 1. Skilled health care provider 2. Outpatient services 3. Inpatient services 4. Surgical services 5. Medication and drugs 6. Laboratory services 7. All of the above are not adequate |
| 2.39 | What kind of services were you or your family offered in the health facility? (More than one answer may apply) | 1. Outpatient services  2. Inpatient Services  3. Referral services |
| 2.40 | Has the CBHI improved the quality of health care? | 1. Extremely improved  2. Partly improved  3. partly deteriorated  4. Extremely deteriorated |
| 2.41 | How certain are you to continue with membership of CBHI | 1. Certainly continue  2. Certain based on some condition’s fulfillment  3. Quite immediately  4. I don’t know  5. I will decide in consultation with my husband |
| 2.42 | Do you think that the benefit packages of the health care services are adequately covered by the CBHI? | 1. Agree that relevant benefit packages are adequately included  0. Relevant packages are not adequately included |
| 2.43 | Do you think the premium contribution to the CBHI is fair? | 0. It is not fair  1. Is fair |
| 2.44 | Do you think that the CBHI decision-making process is transparent? | 0. Not transparent  1. Transparent |
| 2.45 | Do you think that the CBHI decision-making process is inclusive of all relevant actors? | 0. It includes sometimes  1. Inclusive at all times |
| 2.46 | Is advocacy and promotion conducted by the scheme adequate? | 0. It is not adequate  1. It is adequate |
| 2.47 | Do you believe that access to CBHI will improve your access to healthcare services in your community? | 0. No  1. Yes |
| 2.48 | Do you believe that being enrolled in the CBHI plan will make health care more affordable for you and the members of your household? | 0. No  1. Yes |
| 2.49 | From either your experience or understanding, how would you rate the potential financial protection by community-based health insurance against the cost of illness? | 0. None  1. Low to high |
| 2.50 | What do you think is the potential level of access by households to affordable healthcare due to community-based health insurance? | 1. None 2. Low to high |
| 2.51 | How would you rate the potential of community-based health insurance to improve household health consumption patterns by ensuring that healthcare costs are reduced? | 0. None 1. Low to high |
| 2.52 | How would you rate the potential of CBHI to improve the quality of services provided by healthcare givers? | 1. None 2. Low to high |
| 2.53 | How would you rate the potential of CBHI to ensure the constant availability of drugs at health facilities in your community? | 1. None 2. Low to high |
| 2.54 | Is community-based health insurance acceptable to you as a strategy for paying for health care in this area? | 1. Yes 0. No |
| 2.55 | Would you advocate the CBHI to others? | 0. No, I don’t. 1. Yes, I do |

Section III: Wealth index attributes

| S. No | Question | Response | | |
| --- | --- | --- | --- | --- |
| 3.1. | What is the main source of drinking water for members of your household? | Piped water:   1. Piped into dwelling 2. Piped to yard/plot 3. Public tab/standpipe 4. Borehole 5. Dug well: 6. Protected well 7. Unprotected well 8. Protected spring 9. Unprotected spring   Surface water:  River/pond/stream | | |
| 3.2. | What is the main source of water used by your household for other purposes such as cooking and hand washing? | 1. Piped water: 2. Piped into dwelling 3. Piped to yard/plot 4. Public tab/standpipe 5. Borehole 6. Dung well: 7. Protected well 8. Unprotected well 9. Water from spring:   Protected spring  Unprotected spring  Surface water:  River/pond/stream | | |
| 3.3 | Where is that water source located? | In own dwelling  In own yard/plot  Elsewhere | | |
| 3.4 | How long does it take to go there, get water, and come back? | Minutes __________  Don’t know | | |
| 3.5 | In the past two weeks, was the water from this source not available for at least one full day? | Yes  No  Don’t know | | |
| 3.6 | Do you do anything to the water to make it safer to drink? | Yes  No | | |
| 3.7. | What do you usually do to make the water safer to drink? Record all mentioned | Boil  Add bleach/chlorine  Strain through a cloth  Use water filter (Sand/composite/etc.)  Solar disinfection  Let it stand and settle | | |
| 3.8. | What kind of toilet facility do members of your household usually use? | 1. Flush or pour-flush toilet 2. Flush to a piped sewer system 3. Flush to septic tank 4. Flush to a pit latrine 5. Flush to somewhere else 6. Flush, don't know where 7. Pit latrine 8. Ventilated improved pit latrine 9. Pit latrine with slab   Pit latrine without slab/open pit  composting toilet  bucket toilet  Hanging toilet/hanging latrine  No facility/bush/field | | |
| 3.9 | Do you share this toilet facility with other households? | Yes  No | | |
| 3.10 | Including your household, how many households use this toilet facility? | Less than 10  10 or more  Don't know | | |
| 3.11 | Where is this toilet facility located? | In own dwelling  In own yard/plot  Elsewhere | | |
| 3.12 | What type of fuel does your household mainly use for cooking? | 1. Electricity 2. Liquid petroleum gas 3. Natural gas 4. Biogas~~~͠ 5. Kerosene 6. Charcoal 7. Wood 8. Straw/grass 9. Agricultural crop   Animal dung  No food cooked in house | | |
| 3.13 | Is the cooking usually done in the house, in a separate building, or outdoors? | In the house  In separate building  Outdoors  Other specify______________ | | |
| 3.14 | Do you have a separate room which is used as a kitchen? | Yes  No | | |
| 3.15 | Who is the owner of the house? | Me  Rental  Family  Relative | | |
| 3.16 | How many rooms in this household are used for sleeping? | There are --------Rooms | | |
| 3.17 | The main material of the roof of the house? | Natural roofing (no roof, mud, and sod)  Rudimentary roofing (rustic mat/plastic sheet, reed/bamboo, wood planks, and cardboard)  Finished roofing (metal/corrugated iron, wood, calamine/cement, ceramic tiles, roofing shingles) | | |
| 3.18 | The main material of the floor of the house? | Natural floor (Earth/sand, dung)  Rudimentary floor (wood planks, and palm/bamboo)  Finished floor (parquet or polished wood, vinyl or asphalt strips/ plastic tiles, cement, ceramic tiles, carpet) | | |
| 3.19 | Does this household own any livestock, herds, other farm animals, or poultry? | Yes  No | | |
| 3.20 | How many of the following animals do this household own?  If none, record '00'.  If 95 or more, record '95'.  If unknown, record '98'. | Cows bulls_______________  Other cattle______________  Horses____________  Donkeys_______  Mules ____  Camels________  Goats _________  Sheep _________  Chickens/poultry ______  Beehives ___________ | | |
| 3.21 | Do you have separate rooms for cattle? | Yes  No | | |
| 3.22 | Does any member of this household own any agricultural land? | Yes  No | | |
| 3.23 | How many hectares of agricultural land do members of this household own? | ___________hectares | | |
| 3.24 | Does any member of the have any one of these? |  | Yes (1) | No (0) |
|  |  | Electricity  Radio  Television  Non-mobile telephone  Computer  Refrigerator  Table  Chair  Bed with spring matters  Electric mitad  Kerosene  lamp/pressure Lamp-- |  |  |
| 3.25 | Does any member of this household own? |  | Yes (1) | No (0) |
|  |  | Watch  Mobile phone  Bicycle  Motorcycle/scooter  Animal-drawn cart  Car/truck  Boat with motor  Baggage |  |  |
| 3.26 | Does any member of this household have a bank account? | Yes  No | | |
| 3.27 | Does any member of this household have a microfinance account? | Yes  No | | |
| 3.28 | How often does anyone smoke inside your house?  Would you say daily, weekly, monthly, less often than once a month, or never? | Daily  Weekly  Monthly  Less often than once a month  Never | | |

Thank you very much!
